# Supplementary material for: Prevention of tunneled cuffed catheter dysfunction with prophylactic use of a taurolidine urokinase lock: A randomized double-blind trial
Source: PLoS One. 2021 May 20;16(5):e0251793. doi: 10.1371/journal.pone.0251793 (PMC8136626; doi:10.1371/journal.pone.0251793)
Supplement: S2 File — (PDF) [file pone.0251793.s002.pdf]

Clinical Trial Protocol Signature Page

Prevention of tunneled cuffed catheter malfunction with prophylactic use of a taurolidine locking solution containing Urokinase: A prospective and randomized placebo-controlled trial

Appendix A

**Protocol Name:**	Taurolock-Urokinase
**Product:**	TauroLock™U25.000/TauroLock™ HEP500
**Indication:**	Catheter Malfunction
**Protocol Title:**	Prevention of tunneled cuffed catheter malfunction with prophylactic use of a taurolidine locking solution containing Urokinase
**Protocol version and date:**	2.1 - 01/jan/2015
**Sponsor:**	Universitair Ziekenhuis Brussel<br>Dienst Nefrologie<br>Laarbeeklaan 101<br>1090 Brussel
**Coordinating Investigator:**	Dr. F. Bonkain

Coordinating Investigator: ________________________ ______________

Signature of coordinating Investigator Date

____

Printed Name

Principal Investigator of local site: ________________________ ______________

Signature of Principal Investigator Date

____

Printed Name

By my signature, I agree personally supervise the conduct of this study and to ensure its conduct in compliance with the protocol, informed consent, IRB/EC procedures, the declaration of Helsinki, ICH Good Clinical Practice guidelines and the applicable parts of the local regulations governing the conduct of clinical trials

1. Table of Contents

1. Table of Contents 2
2. Roles and Responsabilities 3
3. List of Abbreviations 6
4. Introduction 7
5. Methods 8
   - 5.1 Study aims 8
   - 5.2 Study design 8
   - 5.3 Study population 8
   - 5.4 Study procedures 9
   - 5.5 Flow Chart 12
6. Study medication 13
7. Duration of the study 14
8. Outcomes 14
9. Safety reporting 15
10. Data collection 17
11. Statistical analysis 18
12. Study oversight and ethical issues 19
13. Protocol Revisions 19
14. Insurance 19
15. Conflict of interest statement 19
16. Records Retention at the Trial Site 20
17. Randomization and TCC dysfunction procedures 20
18. List of Tables 23
19. References 26

2. Roles and Responsabilities

F. Bonkain[1], K.M.Wissing[1,2], P. Janssens[1], Freya Van Hulle[1], C. Catalano[2], JC Stolear[3], D.Vandervelde[4], M. Libertalis[5], S. Treille[6], A. Van Craenenbroeck[7], J.F. Fils[8], Ch. Tielemans[1]

[1] Nephrology Department, UZ Brussel VUB
[2] Nephrology Department, CHU Brugmann
[3] Nephrology Department, IMC Tournai
[4] Nephrology Department, Bracops
[5] Nephrology Department, Ixelles
[6] Nephrology Department, CHU Charleroi
[7] Nephrology Department, UZ Antwerpen
[8] Freelance Biostatistician, Ars Statistica, Nivelles

Coordinating Investigator

Florence Bonkain, MD

Universitair Ziekenhuis Brussel, Dept. Of Nephrology

Laarbeeklaan 101, 1090 Brussels, Belgium

Phone: 32 2 477 6055

Fax: 32 2 477 6230

E-mail: florence.bonkain@uzbrussel.be

Sponsor

Universitair Ziekenhuis Brussel, Dept. Of Nephrology

Laarbeeklaan 101, 1090 Brussels, Belgium

Phone: 32 2 477 6055

Fax: 32 2 477 6230

E-mail: florence.bonkain@uzbrussel.be

Study sites and co-investigators

- ***Universitair Ziekenhuis Brussel***
    - Florence Bonkain, MD (Principal and coordinating investigator)
    - Prof. Dr. K.M. Wissing, MD, PhD
    - Prof. Ch Tielemans, MD, PhD
    - Dr P. Janssens, MD

  Dept. of Nephrology
  Laarbeeklaan 101, 1090 Brussels, Belgium
  Phone: 32 2 477 6055
  Fax: 32 2 477 6230
  E-mail: florence.bonkain@uzbrussel.be

- ***CHU Brugmann***
    - Dr. C. Catalano, MD

  Dept. of Nephrology
  Place van Gehuchten 4, 1020 Brussels, Belgium
  Phone: 32 2 477 30 35
  Fax: 32
  E-mail: concetta.catalano@chu-brugmann.be

- ***Centre Hospitalier de Wallonie Picarde - Site IMC Tournai***
    - Dr. J.C. Stolear, MD

  Dept. of Nephrology
  Chaussée de Saint-Amand 80, 7500 Tournai, Belgium
  Phone: 32 69 88 53 75
  Fax: 32
  E-mail: jean-claude.stolaer@chwapi.be

- ***Hôpitaux Iris-Sud, Joseph Bracops***
    - Dr. D. Vandervelde, MD

  Dept. Of Nephrology
  Docteur Huetstraat 79, 1070 Brussels, Belgium
  Phone: 32 2 556 13 01
  Fax: 32
  E-mail: dvandervelde@his-izz.be

- ***Hôpitaux Iris-Sud, Ixelles***
  - Dr. M. Libertalis, MD

  Jean Paquotstraat 63, 1050 Brussels, Belgium
  Phone: 32 2 641 46 18
  Fax: 32
  E-mail: mlibertalis@his-izz.be

- ***CHU Charleroi***
  - Dr. S. Treille, MD

  Dept. of Nephrology
  Boulevard Paul Janson 92, 6000 Charleroi, Belgium
  Phone: 32 71 92 11 31
  Fax: 32
  E-mail: serge.treille@chu-charleroi.be

- ***Universitair Ziekenhuis Antwerpen***
  - Dr.A. Vancraenenbroeck, MD

  Dept. of Nephrology
  Wilrijkstraat 10, 2650 Edegem, Belgium
  Phone: 32 3 821 34 35
  Fax: 32
  E-mail: amaryllis.vancraenenbroeck@uantwerpen.be

<u>Study Statistician</u>

J. F Fils

Freelance Biostatistician, Ars Statistica

Boulevard des Archers, 40

1400 Nivelles

3. List of Abbreviations

TCC: Tunneled cuffed catheters

HD: Hemodialysis

Rt-PA: Recombinant tissue plasminogen activator

DVT: Deep venous thrombosis

ITT: Intention- to-treat

PD : Peritoneal dialysis

4. Introduction

The use of tunneled cuffed catheters (TCC) has two main complications, catheter malfunction due to thrombosis and catheter-related bacteremia. These complications cause high morbidity and mortality[1] among hemodialysis (HD) patients, and generate high costs. In order to reduce the risk of TCC-related malfunction and bacteremia, a locking solution is instilled into the dead space of the TCC after each HD session. Many locking solutions have been developed (heparin, trisodium citrate associated or not with antibiotics) but currently the most efficient and secure locking solution remains a matter of debate.

In spite of the use of locking solutions the average one-year survival reported for TCC is 50% with up to two thirds of the failures due to thrombotic complications[2,3]. A recent prospective and randomized study has shown that the prophylactic use of recombinant tissue plasminogen activator (rt-PA) once weekly reduces the incidence rate of TCC malfunction and bacteremia as compared to the exclusive use of heparin as locking solution[4]. However, the systematic use of thrombolytic agents is costly, as it implicates the regular administration of thrombolytic agents to patients who would have never developed TCC malfunction. A more cost-effective approach might be to restrict the use of prophylactic thrombolytic agents to patients with a history of thrombotic TCC malfunction.

Currently, our policy for TCC management consists to use a routine locking solution containing taurolidine, citrate 4% and heparin (Taurolock™ HEP 500, Tauro-Implant, Winsen, Germany – www.taurolock.com) for TCC. In case of TCC thrombotic malfunction (defined in the Methodology and the Appendix section), Urokinase (Actosolv™, Eumedica, www.eumedica.com) is employed as fibrinolytic agent to restore the patency of the TCC.

The objective of our study is to investigate whether the substitution of the standard locking solution with a locking solution containing taurolidine and Urokinase once a week (Taurolock ™ U 25,000 - www.taurolock.com) reduces the rate of TCC thrombotic dysfunction in hemodialysis patients with a history of malfunction requiring Urokinase therapy. The hypothesis that use of the Urokinase containing locking solution will reduce the incidence of TCC thrombotic malfunction by 50% will be assessed in a multicenter, randomized, double-blinded controlled trial.

5. Methods

5.1 Study aims

The primary objective of the study is to determine whether substituting Taurolock™ HEP 500 by Taurolock ™ U 25,000 once per week, compared to using Taurolock™ HEP 500 after each dialysis session as a locking solution will decrease the incidence of TCC thrombotic dysfunction requiring thrombolytic locking solution in a population of hemodialysis patients with a history of TCC dysfunction by at least 50%.

The secondary objectives are to determine whether substituting Taurolock™ HEP 500 by Taurolock ™ U 25,000 once per week as a catheter locking solution will decrease

- the incidence of TCC removal (for any reason – TCC thrombosis as well as TCC-related bacteremia)
- the use of systemic thrombolytic therapy for thrombotic TCC dysfunction
- the incidence of TCC-related bacteremia and exit-site infection

An additional objective of the study is an analysis of the cost-effectiveness of Taurolock ™ U 25,000 as compared to standard therapy that will be detailed in a separate protocol.

As pre-specified subgroup analysis, the study will also test whether gender, history of previous venous thromboembolism (DVT, pulmonary embolism and/or AVF/graft failure due to thrombosis) and use of anticoagulation or antiplatelet therapy have an effect on the efficacy of Taurolock ™ U 25,000 in the prevention of TCC related thrombotic dysfunction.

5.2 Study design

This is a prospective, multicenter, randomized, placebo-controlled trial with blinding of patients, health care providers and all study staff

5.3 Study population

Inclusion criteria

The study will include adult prevalent patients undergoing HD at least three times a week via a TCC since more than 3 months. To be eligible the current TCC must have been dysfunctional with requirement for therapeutic thrombolytic therapy with Urokinase (as locking solution) for at least two separate episodes of TCC thrombotic dysfunction during the 6 months before inclusion as defined in the appendix of this protocol.

Inclusion into the trial is only permitted if the last therapeutic thrombolytic locking solution was administered more than three dialysis sessions (one week) ago. Patients will be recruited at 7 Belgian dialysis Units (High-Care as well as Low-care dialysis Units). All patients have to provide written informed consent prior to inclusion into the study.

Exclusion criteria

Exclusion criteria are a history of heparin-induced thrombopenia, presence of a femoral TCC, established intolerance for the taurolidine locking solution (citrate, taurolidine) or for Urokinase, current administration of antibiotics for TCC related infection (exit site or bacteremia) during the week before the inclusion and scheduled holidays for more than 2 weeks during the study period. Patients with TCC dysfunction due to mechanical problems such as kinking, TCC migration, misplacement, inadequate length and patient malposition are also excluded.

5.4 Study procedures

Screening for eligible patients

Participating centers will screen their population of hemodialysis patients treated with a tunnelled catheter for those having received at least two therapeutic interventions with Urokinase locking solutions during the last 6 months. A Urokinase treatment consists in the administration of at least one to maximum of three locks during successive dialysis sessions after diagnosis of thrombotic catheter dysfunction. The different treatments during the 6 month period have to be separated by at least three dialysis sessions during which catheter function has been restored by the previous treatment. The last Urokinase treatment has to be administered at least 3 sessions (one week) before starting the inclusion process.

Patients fulfilling the requirements in terms of inclusion/ exclusion criteria and who provide written informed consent begin a screening process of three successive dialysis sessions during which the TCC mean blood flow and the absence of a TCC-related infection are verified. Patients are informed that inclusion into the trial depends on the results of the screening process.

Eligible patient can be included into the trial in case of fulfilment of the following requirements during the screening period:

- mean TCC blood flow is higher than 250 ml/min with pre-pump arterial pressure > -250 mmHg and post-pump venous pressure < +250 mmHg during each dialysis session
- no thrombolytic locking solution (Urokinase) is used
- absence of TCC-related infection (exit-site infection or bacteremia).

Patient with TCC-related infection could be included, in case of TCC has not to be removed, once infection has been treated successfully and after the patient has not received antibiotics for at least 3 dialysis sessions.

In the same way, patients with catheters who initially did not reach the attended minimal TCC blood flow or /and needed Urokinase during the screening process, could be in a second time rescreened and included if all the criteria are at this time fulfilled. All patients fulfilling the inclusion criteria in terms of thrombotic dysfunction and Urokinase treatments will be recorded in a screening log.

Reasons for screening failure or other causes of non-inclusion will be recorded in the log according to the latest CONSORT guidelines.

Determination of baseline catheter function

Potential candidates have to be screened to evaluate baseline TCC function. Baseline blood flow is defined as the mean of the average blood flows during 3 consecutive dialysis sessions of the screening period. The mean blood flow of each dialysis session is calculated as the total blood volume processed in millilitres divided by the time on dialysis in minutes.

Inclusion procedure

For each eligible patient the sub-investigators at the local centres will send an e- mail or a fax to the principal investigator and the clinical nurse of the UZ Brussel hospital with patient ID, the center ID and confirmation that inclusion and exclusion criteria are fulfilled and the informed consent is signed.

Randomization procedure

Patients will be allocated either to the intervention or to the control group using block randomization with permuted blocks of four stratified for the participating centres. Sealed allocation envelopes will be prepared centrally at the UZ Brussel by the hospital pharmacist who will also provide the study medication to the participating centres. The detailed randomization procedure is provided as an appendix to the present protocol.

Blinding procedure

The allocation is blinded for everyone involved in the study. The producer will deliver to the central pharmacy of the UZ Brussel hospital for each included patient the blinded locking solution which will be administrated once weekly at the end of the dialysis session before the longest interdialytic interval (i.e. typically on Friday or Saturday) for 6 months. The Taurolock ™ U 25,000 consists on a separate vial of Urokinase destined to be dissolved with the content of an ampoule containing taurolidine and citrate 4% just before administration. The placebo group will receive a catheterlock from an identical-looking vial without Urokinase and TauroLock-HEP500 from an identical-looking ampoule. The vial in the placebo group will include the excipients of the commercial product but no Urokinase. The excipients (glycin, etc.) have no known effect on the product after mixing with TauroLock-Hep500. The appearance of all the vials and the ampoules is identical for the two groups. Together with the study medication, a sealed envelope with the unblinding code will be shipped to the study site.

If a medical emergency occurs and a decision regarding the subject's condition requires knowledge of the treatment assignment, the study blind may be broken for the specific subject. Whenever possible, the investigator should consult with the responsible investigator of the trial prior to unblinding any subject. Any broken blind will be clearly justified and explained by a comment in the CRF.

Withdrawal from the study

Patients can withdraw from the study either by withdrawal of consent or by request from the investigator.

Removal of catheter secondary to any cause, treatment of catheter thrombosis with systemic thrombolytic therapy, withdrawal of consent, renal transplantation, use of an AVF or a graft, transfer to PD or to another center, patient death and loss to follow up exclude the patient from further follow up in the study.

In case the study medication is stopped although the patient is continuing hemodialysis treatment with the catheter, data concerning study outcomes should continue to be collected in order to allow analysis of the patient according to the intention to treat principle.

Systemic thrombolytic therapy for thrombotic catheter dysfunction

The participating hemodialysis units have different approaches to manage TCC thrombotic dysfunction that is resistant to three consecutive administrations of therapeutic Urokinase locks. Some centres change the dysfunctional TCC while other centres administer systemic thrombolysis in case clotting around the catheter tip was shown after opacification of the superior vena cava. To avoid bias in the follow up of patients in different centres, the administration of systemic thrombolysis is considered as an intervention equivalent to catheter replacement and terminates follow up in the study.

5.5 Flow Chart

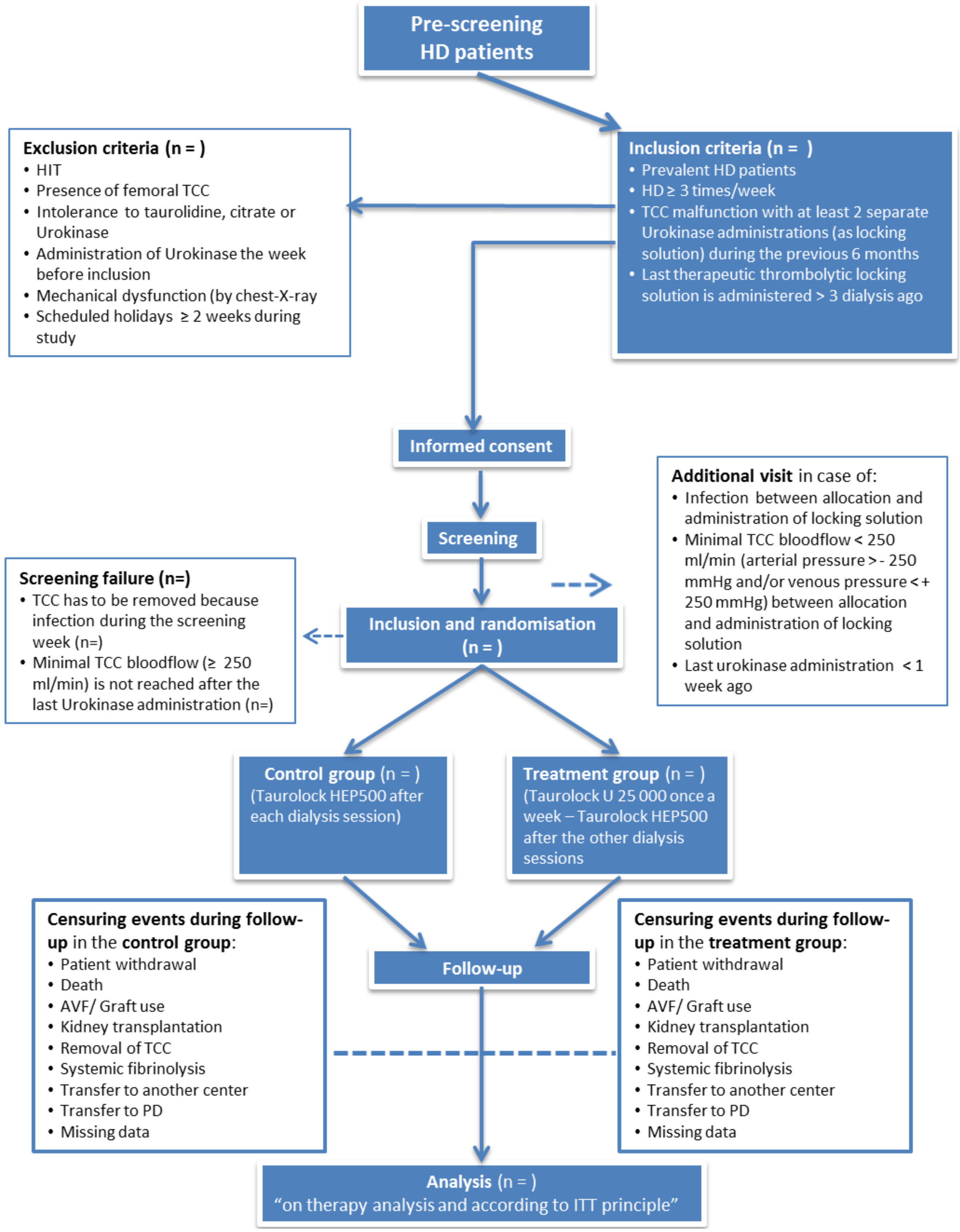

Abbreviations: HD: hemodialysis ; TCC: tunneled-cuffed catheters ; AVF: arterio-venous fistulae; PD: peritoneal dialysis; ITT: intention-to-treat

6. Study medication

Administration of the study medication

Physicians, dialysis nurses, patients and study coordinators of each center are blinded for the allocation. When a new patient is included, the hospital pharmacy of the Universitair Ziekenhuis Brussel will receive the center and patient identifiers via e-mail and will prepare and dispense to the participating centers blinded locking solutions sufficient for 6 months of treatment. For patients allocated to the intervention arm the Taurolock U 25,000 will be administered once weekly on a fixed day i.e. the day before the longest dialysis-free interval (Friday or Saturday depending on the patients schedule). The TauroLock-Hep500 administered after the first and second dialysis session of the week will be for both treatment and placebo group the regular commercially available product with commercial labeling. After the third dialysis session of the week, the patients in the treatment arm will receive Taurolock U 25,000. Urokinase will be delivered in a separate vial and dissolved with the content of an ampoule containing citrate and taurolidine just before use. The control group will receive an identical placebo powder containing the excipients of the Taurolock U 25,000 without Urokinase. This placebo powder has to be dissolved in the classical Taurolock HEP500 locking solution. The excipients (glycin, etc.) have no known effect on TauroLock-Hep500. If a patient needs an extra dialysis session he will receive the classical locking solution (Taurolock HEP500) from the stock of the center at the end of that session.

Storage of the study medication

The study medication should be stored at 15-25°C**.** Long term storage over weeks in an environment warmer than 25°C is not allowed .The storage room temperature should be monitored in each participating center. In case of deviations, the principal investigator should be informed and will disqualify the study product from the trial. Short term storage (some hours) between 25°C – 30°C will not significantly affect the activity of the product.

Labeling of the study medication

After all inclusion criteria are met, the pharmacist of the central site (UZ Brussel) is informed and he performs the allocation. A kit number shall be assigned to the patient and this number will be the same for 6 months. The principal investigator and pharmacist at the local site shall be informed of this kit number by mail by the pharmacist of UZ Brussel. The box with sealed envelope will be shipped to the site. The sealed envelope contains the information of allocation and should only be opened in case of emergency. Each box for one patient contains 6 boxes (6X5= 30 doses) blinded product with same kit number for max. 30 weeks. Each vial and ampoule is labeled and the removable label should be pasted on the medication log form for each patient, completed with the date of administration.

Drug Accountability

Upon receipt, the investigator is responsible for taking an inventory of the study product. A record of this inventory must be kept and usage must be documented on study agent inventory forms provided by the sponsor.

Study agent inventory forms will be examined and reconciled by an unblinded monitor or designee. At the end of the study, all used and unused medication must be accounted for a study agent accountability form.

7. Duration of the study

The duration of the study is 6 months starting from the moment of randomization.

8. Outcomes

The **primary outcome** is the median number of TCC thrombotic malfunction requiring therapeutic thrombolytic locking solution at the end of the follow-up.

As pre-specified subgroup analysis, the study will also test whether gender, history of previous venous thromboembolism (DVT, pulmonary embolism and/or AVF/graft failure due to thrombosis) and use of anticoagulation or antiplatelet therapy have an effect on the efficacy of Taurolock ™ U 25,000 in the prevention of TCC related thrombotic dysfunction.The present study does not take into account the recommendations of the KDOQI guidelines defining a TCC-related dysfunction as a blood flow less than 300 ml/min[2] (with prepump arterial pressure not less than -250 mmHg and a venous pressure not more than +250 mmHg). Blood flow lower than 300 ml/min is commonly used in Europe (with the same target single-pool Kt/V of 1.2) because dialysis sessions are longer. We preferred to define TCC-related dysfunction as a change in the access flow observed for each patient.

The present protocol defines thrombotic TCC dysfunction as the occurrence of one or more of the following events:

- The occurrence during 2 consecutive dialysis sessions of a mean blood flow decreased by ≥15% as compared to the average blood flow measured during the three dialysis sessions before inclusion, and not resolved by patient repositioning or TCC flushing*, and persistent after switching of the arterial and venous blood lines. Blood flow has to be maintained with pre-pump arterial pressure superior to -250 mmHg and/or post-pump venous pressure lower than +250 mmHg.
- The inability to initiate dialysis due to complete occlusion of at least one line of the catheter (late manifestation)*

*According to the 2006 KDOQI Guidelines

In case of an accidentally missed Urokinase administration, the patient should receive the therapeutic Urokinase on the next dialysis session, independently of the mean blood flow observed on this moment.

The following **secondary outcomes** will be assessed

- Removal of the TCC (assessed as proportion of patients with removal of the catheter and as time to removal of the catheter). Effect of the intervention will be assessed for catheter removals for any cause as well as for removals due to thrombosis or infection.
- Proportion of catheters receiving systemic thrombolytic therapy because of thrombosis resistant to treatment with therapeutic Urokinase locking solution.
- Catheter-related bloodstream infections (according to CDC definitions- see table 1)[2,5].
  No blood samples are to be systematically obtained for bacteriologic analyses during the study. Only patients with fever, chills, and/or biological inflammatory syndrome are to be evaluated by blood cultures. Data of TCC exit-site infection will also be collected.

The **third outcome** is a cost-effective analysis of both procedures. This analysis will compare the total cost of locking solutions and therapeutic use of Urokinase for catheter dysfunction in both arms of the study in order to assess whether the use of Taurolock ™ U 25,000 once a week in addition to Taurolock™ HEP 500 is cost effective due to a reduction in the use of therapeutic Urokinase. This analysis will be detailed in a separate protocol.

9. Safety reporting

The study investigators have the responsibility to perform

1. the safety reporting of a medical device
2. to report the SAE's and complete the SAE document in the investigator's binder

9.1. Safety reporting of a medical device

As Taurolock Urokinase is a medical device, the European guidelines (clinical investigations: serious adverse event reporting, under directives 90/385/EEC and 93/42/EEC) are applicable.

9.1.1 Definitions

Adverse **D**evice **E**ffect (**ADE**) (Definition –IS0/FDIS 14155)

Adverse event related to the use of an investigational medical device. This includes any adverse event resulting from insufficiencies or inadequacies in the instruction for use, the deployment, the implantation, the installation, the operation, or any malfunction of the investigational medical device. This includes any event that is a result of a use error or intentional misuse.

9.1.2 What should be notified?

Any dysfunction or any change of the characteristics and/or performance of a device, and inadequacy in the labeling or instructions, which might lead to or have led to death or serious relapse in the state of health of a patient , a user or a third party.

Any technical or medical reason related to the characteristics or performance of a device for reasons shown in the previous paragraph and having led to the systematic withdrawal from the market by a manufacturer of devices of the same type.

9.1.3 Who should notify?

Not only the manufacturers or their representatives but also persons distributing devices, notified bodies, practitioners and people responsible for receiving and/or delivering devices should all signal incidents to: Federal Agency for Medicines and Health Products-Vigilance Division: meddev@fagg-afmps.be

A copy of the incident must be forwarded to Taurolock@uzbrussel.be or faxed to 32 2 477 6220.

9.1.4 When must incidents be notified?

Incidents must be notified as quickly as possible using the quickest means possible. Incidents that have led to death or serious injury must be notified immediately.

9.1.5 How should be notified?

The notification documents are available on sites:

Dutch Speaking Sites : see materiovigilantie: *"Formulier voor de melding van en incident met een medisch hulpmiddel" PDF 227.62Kb*

http://www.faggafmps.be/nl/MENSELIJK_gebruik/gezondheidsproducten/medische_hulpmiddelen_hulpstukken/materiovigilantie

French Speaking Sites: see matériovigilance: *"Formulaire de notification d'incident avec un dispositive medical" PDF 431.19Kb*

http://www.faggafmps.be/fr/humain/produits_de_sante/dispositifs_medicaux/materiovigilance

9.2 Safety reporting of SAE

A SAE includes any event that results in any of the following outcomes :

1. Death
2. Life-threatening, ie, the subject was, in the opinion of the investigator, at immediate risk of death from the event as it occurred.
3. Persistent or significant incapacity or substantial disruption of the ability to conduct normal life functions
4. Requires in-patient hospitalization or prolongs hospitalization
5. Congenital anomaly/birth defect
6. Other medical significant events that, based upon appropriate medical judgment, may jeopardize the subject and may require medical or surgical intervention to prevent one of the outcomes listed above, eg, allergic bronchospasm requiring intensive treatment in an emergency room.

Subjects experiencing SAE's should be followed clinically until their health has returned to baseline status, or until all parameters have returned to normal, or have otherwise been explained. It is expected that the investigator will provide or arrange appropriate supportive care for the subject.

All SAE's should be completed in the SAE document in the SAE section of the investigator's site file on site.

10. Data collection

Baseline data and ongoing data collection will be recorded after each dialysis session by the local investigators in a patient record book (table 2 and 3).

For each included patient, the mean blood flow has to be calculated at the end of the dialysis session (within the 5 last minutes as the ratio of the blood volume treated / dialysis time in minutes) and to be compared to the baseline TCC function to evaluate the need of Urokinase administration.

A fax mentioning the relevant parameters of dialysis (mean blood flow, use of Urokinase, TCC-related infection or systemic fibrinolysis) has to be sent two times per month to the PI in order to check protocol adherence.

Data will be recorded from study entry until study completion (6 months) or until the occurrence of a censuring event (patient's death, use of AVF, transfer to another HD centre, transfer to peritoneal dialysis, kidney transplantation, systemic fibrinolysis or removal of the TCC).

UZ Brussel or the representative shall have the right to inspect on a regular basis the included patient's data in the participating sites.

Patients who decide to be dialyzed in another center during the study will be censored for the period they are not receiving the study medication as the outcome of interest cannot be adequately assessed while the patient is dialysed in centres that are not participating in the study.

11. Statistical analysis

Statistical hypothesis

We arbitrarily considered a 50% reduction of the use of Urokinase as clinically relevant. A review of patients with at least 2 therapeutic Urokinase treatments over a 6 months period showed that these patients had a median of 3 Urokinase treatments over 6 months (range 2-9). We expect a median of 2 Urokinase treatments over 6 months (range 1-5) with the Taurolock™ HEP 500.

Sample size calculation for rank data with ties (Zhao, Rahardja & Qu, 2008) indicates that, considering a drop-out rate of 20%, with a type I error of 5% and a power equal to 80%, 46 patients in total are needed (23 patients by group) in order to recruit 18 valuable patients by groups in total.

Considering a drop-out rate of 20%, with a type I error of 5% and a power equal to 90%, 64 patients in total are needed (32 patients by group) in order to recruit 25 valuable patients by groups in total.

In case more than 64 patients could be included into the trial, the statistical hypothesis will be modified during the trial by an amendment of the protocol in order to obtain a more precise estimate of the 95% confidence interval of the effect size.

The primary outcome is expressed as number of catheter dysfunctions requiring Urokinase treatment. Hypothesis testing will be done by Wilcoxon rank sum test.

To take into account differences in time at risk due to catheter removal and patient drop-out in the two arms of the study, the primary outcome will also be expressed as number of catheter dysfunctions requiring Urokinase treatment per 100 patient years at risk and will be expressed with 95% confidence intervals. Time at risk for calculation of the incidence rate of catheter dysfunction is defined from inclusion to either end of the 6 month follow up period or the occurrence of a censuring event (TCC removal, treatment of catheter dysfunction with systemic thrombolytic therapy, patient's death, change of HD center, use of AVF, shift to peritoneal dialysis or kidney transplantation). The effect of the intervention will be expressed by the rate ratio with 95% confidence interval. Hypothesis testing will be done by Poisson regression or eventually by negative binominal regression modeling in case the distribution of outcomes does not follow the Poisson distribution. These regression models also allow hypothesis testing and calculation of rate ratios after adjustment for covariates.

Survival free of the primary endpoint will be calculated by the Kaplan-Meier method with 95% confidence estimates. Hypothesis testing will be done using the log-rank test.

All patients with available data will be analyzed according to the intention-to-treat principle. Time at risk for the development of the outcomes of interest stops at removal of catheter secondary to any cause, treatment of catheter thrombosis with systemic thrombolytic therapy, withdrawal of consent for collection of data, renal transplantation, patient death or loss to follow up. The effect of the intervention on the primary and secondary outcomes will also be analysed in an "on therapy"

population. Patients in whom the study medication was not administered for more than two consecutive weeks will be excluded from the "on therapy" analysis.

12. Study oversight and ethical issues

We conduct this study in accordance with the ethical principles of the Declaration of Helsinki, according to the rules and guidelines of Good Clinical Practice (GCP) and in line with local regulatory requirements.

The study is to be approved by the ethics committee at each participating centre. The ethical committee of the Universitair Ziekenhuis Brussel will act as central ethical committee.

The first author designed and supervised the trial and the statistical analysis plan in collaboration with the staff of the UZ Brussel, Brussels. One co-investigator per dialysis Unit was designated for the other participating centres. The first author wrote the first draft of the study protocol; subsequent drafts were reviewed by the others co-investigators. The co-investigators certify that the study will be performed in accordance with the protocol and vouch for the accuracy and completeness of the reported analyses.

The informed consents have been written in Dutch and French. In order to participate to the study all patients will have to give informed consent. Patients may withdraw from the study at any time without prejudice tot their future care.

13. Protocol Revisions

Protocol amendments will be prepared and approved by the principal investigator of the trial. All protocol amendments will be signed by the investigator and submitted to the IRB/IEC for review, prior to implementation.

14. Insurance

An insurance policy was taken out to cover any potential risks associated with the intervention. The sponsor will provide the investigator with a copy of it. Ethias: nr polis 45.145.223

15. Conflict of interest statement

The investigators have no conflict of interest in relation with the present study.

Tauro-Implant provides the study medication and the corresponding placebo free of charge to the patients participating in the trial. Tauro-Implant does not provide additional funds for the realization of the trial, has not participated in the writing of the study protocol and has no role in the collection of data, as well as analysis, interpretation and presentation of the results.

None of the investigators has received funding form Tauro-Implant for work realized in the context of the present clinical trial.

16. Records Retention at the Trial Site

The investigator will ensure that the trial site file (investigator's binder) is maintained in accordance with section 8 of the ICH GCP guidelines and as required by applicable local regulations. The investigator/institution will take measures to prevent accidental or premature destruction of these documents.

The participating site shall keep, complete, and maintain accurate and authentic accounts, notes, technical reports, data, information and records of the work performed, including case report forms ("data") during 30 years. Sponsor or its representative shall have the right to inspect and make copies of such data during regular business hours.

17. Randomization and TCC dysfunction procedures

a. Procedure for stratified randomization in commuted blocks of four using the Excel random number generator for the Taurolock study

Separate random sequences of inclusion into the Taurolock study for each center (stratification for center) with commuted blocks of four will be prepared using the Excel random number generator according to the technique previously reported by Steve Simon (7). Stratification is used to ensure that each center includes approximately the same number of patients in the placebo and Urokinase patients to avoid the introduction of bias in center-specific management of TCC that are unrelated to the intervention of interest.

Definition of the centers

1. UZ Brussel
2. CHU Brugmann
3. IMC Tournai
4. Iris Sud Bracops
5. Iris Sud Ixelles
6. CHU Charleroi
7. UZ Antwerpen

Additional randomization lists can be generated by the same procedure in case other centers will participate in the study.

Definition of the variable columns in Excel and Generation of the final randomization lists for each center

Randomization will be done with 1:1 allocation in the intervention and control arm.

Randomization lists of 20 patients will be generated for each participating center.

Four columns are generated in an Excel sheet:

1. Sequence (1-20)
2. Group: 10 Placebo and 10 Urokinase
3. Block: For 20 patients 5 blocks of 4 patients are required
4. RandNum: Generated for each line by the "=RAND()" function in Excel

The definitive randomization sequence can then be generated by sorting the columns "Group", "Block" and "RandNum" by first "Block" and then "RandNum". Sequence is not to be included in the sorting procedure to keep the sequence order from 1 to 20.

The randomization sequences for all centers participating in the study are generated by the pharmacist Mr. Nils Noppe. All other persons implicated in the conduct of the study will remain blinded as to the allocation sequence.

The final randomization lists have to be conserved as an Excel file and as print-out hard copies in the UZ Brussel Pharmacy by Mr. Noppe.

These lists serve to prepare the randomization envelopes with the patient identifiers defined by center id in combination with sequence number. Example Center 1 -Patient 001.

Once a new patient is randomized Mr. Noppe prepares the corresponding study medication which is forwarded to the investigators by the responsible hospital pharmacy.

Additional procedures

For the improbable situation that a single center includes more than 20 patients a new randomization list for the center can be generated by the same procedure defining sequence numbers 21-40.

The procedure also allows the generation of additional lists in case new study sites are willing to join the study.

b. Definition of a tunneled cuffed dialysis catheter (TCC) dysfunction (at least one criterion has to be present):

- Blood flow decreasing by ≥15% during 2 consecutive dialysis sessions as compared to the average blood flow during the screening visits, persisting after flushing of TCC, repositioning of the patient and/or switching of the arterial and venous blood lines
- Inability to initiate dialysis due to complete occlusion of at least one line of the catheter (late manifestation)*

*According to the KDOQI guidelines

c. Protocol for management of thrombotic TCC dysfunction

In case of thrombotic dysfunction, we have a step-by-step protocol:

1) a saline flushing (NaCl 0.9%) may restore the permeability in several cases

2) If the problem is not resolved: Urokinase (50,000 IU per lumen) is injected as locking solution in the dead space of each lumen of the dysfunctional TCC. Injection can either occur at the end of the HD session as an interdialytic lock or before starting dialysis treatment in case initiation of treatment was impossible because of catheter thrombosis. Under these conditions the lock remains in place for 20 minutes before dialysis session is initiated. We choose to administer in each case of TCC dysfunction anyway 3 consecutive Urokinase locking solutions (considered as one thrombotic event)

3) In case of recurrent dysfunctions of TCC, an opacification is performed in order to identify the source of the problems and a collegial discussion is planned with our interventional radiologists to solve the problem. The choice to remove the catheter or to perform a systemic thrombolysis is made by the nephrologist, based on the personal clinical experience. In case of replacement of the catheter or use of systemic thrombolytic therapy the catheter reaches the endpoint of the study

4) In any case, if the blood flow is not restored and if an HD session is mandatory, a temporary femoral catheter is placed.

18. List of Tables

Table 1: Definition for TCC-related bacteremia

**Definite bacteremia**	**Probable bacteremia**
- Septic thrombophlebitis with a single positive blood culture, or<br>- Single positive blood culture and positive culture of catheter segment with identical organism, or<br>- 10-fold colony count difference in blood cultures drawn from catheter and peripheral blood, or<br>- Single positive blood culture and positive culture from discharge or aspirate from exit-site, tunnel or pocket with identical organism<br>- in a symptomatic patient with no other apparent source of infection.	- Two or more positive blood cultures with no evidence for source other than catheter, or<br>- Single positive blood culture for S. aureus or Candida with no evidence for source other than catheter, or<br>- Single positive blood culture for coagulase negative Staphylococcus, Bacillus, Corynebacterium, Enterococcus in immunocompromised or neutropenic patients or in patients receiving TPN with no evidence for source other than catheter defervescence of symptoms after antibiotic therapy with or without removal of catheter, in the setting in which blood cultures confirm infection, but catheter tip does not (or catheter tip does, but blood cultures do not) in a symptomatic patient with no other apparent source of infection.

Table 2: Baseline characteristics of the patient population and ongoing data collection

Baseline data :
- Age, sex, origin, duration on dialysis, cause of renal failure
- Type of TCC, date of placement, localisation, history of TCC removal for thrombosis or for bacteremia<br>- History of AVF/graft failure due to thrombosis
- Comorbidity (diabetes, hypertension, ischemic cardiomyopathy, peripheral arteriopathy, active neoplasm, venous thromboembolism : DVT, pulmonary embolism)
- Medication use: anticoagulation, antiplatelet agent
- Hemodialysis modality, time on dialysis (min), weight loss, target weight
- EPO dose per week (UI/kg/week)
- Anticoagulation on dialysis, mean heparine dose
Monthly laboratory data :
- Hemoglobine, platelets, albumin, CRP
- Monitoring of dialysis efficacy (URR and single pool Kt/V)

Table 3: Study period table

Parameter	(Pre)-Screening	Optional addit visit	Month 1			Month 2			Month 3			Month 4			Month 5			Month 6		
**A: every month**<br>**B: each dialysis session**<br>**C: every week (Fri or Sat)**			**A**	**B**	**C**	**A**	**B**	**C**	**A**	**B**	**C**	**A**	**B**	**C**	**A**	**B**	**C**	**A**	**B**	**C**
**General data**																				
Demographical (age, sex)	X																			
Verify Baseline data	X																			
Calculate mean of the average blood flow during the 3 screening dialysis sessions	X	X																		
Verify Inclusion/Exclusion	X	X																		
Informed consent	X																			
Randomisation	X																			
Blood volume treated in ml	X	X		X			X			X			X			X			X	
Dialysis time in min	X	X		X			X			X			X			X			X	
Mean blood flow (Qb) during dialysis session (blood volume/time on dialysis) in ml/min	X	X		X			X			X			X			X			X	
Use of Urokinase and reason why	X	X		X			X			X			X			X			X	
Presence of catheter – related bacteremia/exit-site infection	X	X		X			X			X			X			X			X	
Systemic fibrinolysis therapy				X			X			X			X			X			X	
Removal of the TCC and reason why				X			X			X			X			X			X	
Holiday<br>(date departure and return)				X			X			X			X			X			X	
**Lab analysis**																				
Hemoglobin	X		X			X			X			X			X			X		
Platelets	X		X			X			X			X			X			X		
Albumin	X		X			X			X			X			X			X		
CRP	X		X			X			X			X			X			X		
**Monitoring of dialysis efficacy**																				
URR	X		X			X			X			X			X			X		
Single pool Kt/V	X		X			X			X			X			X			X		
**Administration of study medication**					X			X			X			X			X			X

19. References

1. Di Iorio BR, Bellizzi V, Cillo N et al. Vascular access for hemodialysis: the impact on morbidity and mortality. J Nephrol. 2004;17(1):19-25.

2. National Kidney Foundation. Vascular Access Work Group . Clinical Practice Guidelines for Vascular Access . Am J Kidney Dis . 2006;48(7)(suppl 1):S176–S247

3. Trerotola SO, Johnson MS, Harris VJ, et al. Outcome of tunneled hemodialysis catheters placed via the right internal jugular vein by interventional radiologists. Radiology 1997;203:489-495

4. Hemmelgarn BR, Moist LM, Lok CE et al. Prevention of Dialysis Catheter Lumen malfunction with recombinant tissue plasminogen activator. N Engl J Med. 2011;364(4):303-312.

5. O'Grady NP, Alexander M, Dellinger EP, et al. Guidelines for the Prevention of Intravascular Catheter-Related Infections. Centers for Disease Control and Prevention. MMWR Recomm Rep. 2002; 9 (51): 1-29

6. Zhao Y.D., Rahardja D., Qu Y. (2008). Sample size calculation for the Wilcoxon-Mann-Whitney test adjusting for ties. Statistics in Medicine, 27, 462-468.

7. Simon S. (1999) A simple approach for randomisation. BMJ 1999; 319:703 (doi: http://dx.doi.org/10.1136/bmj.319.7211.703)
