# Supplementary material for: Prevention of tunneled cuffed catheter dysfunction with prophylactic use of a taurolidine urokinase lock: A randomized double-blind trial
Source: PLoS One. 2021 May 20;16(5):e0251793. doi: 10.1371/journal.pone.0251793 (PMC8136626; doi:10.1371/journal.pone.0251793)
Supplement: Attachment — Submitted filename: Bonkain_PlosOne_response_reviewers.docx [file pone.0251793.s003.docx]

**PONE-D-20-30158
Prevention of tunneled cuffed catheter dysfunction with prophylactic use of a taurolidine urokinase locks: a randomized double-blind trial**

Dear Dr Barretti,

We would first like to thank the editor and reviewers for taking the time to read the study and provide their comments. We are very grateful for the opportunity to publish this study in your journal.

Please find below answers to your questions or comments:

**Editor comments :**

**1. Please ensure that your manuscript meets PLOS ONE's style requirements, including those for file naming.**

We confirm that PLOS ONE’s style requirements have been respected in the manuscript.

**2. During your revisions, please note that a simple title correction is required: the title in the manuscript file describes "use of a taurolidine urokinase lock", whereas the submission site describes "use of a taurolidine urokinase locks"**

Title of the manuscript has been changed to "use of a taurolidine urokinase lock" in the manuscript and in the title given in the online submission web application.

**3. To comply with PLOS ONE submission guidelines, in your Methods section, please provide additional information regarding your statistical analyses.**

The authors have made considerable efforts to conduct the study according to the highest methodological standards. The study protocol and statistical analysis plan have been previously published as a separate paper (Reference 31 of the manuscript).

The full dataset (after complete anonymization) as well as the Stata “do” file with all the statistical analysis that were realized are publicly available in the Zenodo data repository (<https://doi.org/10.5281/zenodo.4514859>). The analysis reported in the paper are therefore fully reproducible by the interested reader.

At the request of the editor and following the reporting guidelines of PlosOne we have added additional information on the treatment of outliers and missing values. It is now clearly stated that all hypothesis testing was two-sides with a type I error of 0.05 to reject the null hypothesis. We have also reported in detail the statistical methodology used for the additional analysis requested by the reviewers and which were not part of the initial statistical analysis plan. The type and version of the software used for the analysis has also been added (Page 11).

**4. Please amend your current ethics statement to include the full name of the ethics committee/institutional review board(s) that approved your specific study.**

The names of the local institutional review boards of the participating centers which all approved the study have been added to the ethics statement in the methods section (Page 6)

**5. We note that you have stated that you will provide repository information for your data at acceptance.**

The full dataset as well as the STATA do file of the analysis as well as a description of the variables and data labels have been uploaded to the Zenodo data registry (<https://doi.org/10.5281/zenodo.4514859>).

To preserve patient confidentiality the dataset has been anonymized removing all patient related dates, the dialysis center, patient initials and the gender (before upload). A note on the methodology used for anonymization has been added to the description of the dataset.

**6. Please include captions for your Supporting Information files at the end of your manuscript, and update any in-text citations to match accordingly.**

Caption for Supporting Information (S1 Fig. CONSORT 2010 Checklist- see page 34) has been added at the end of the manuscript as requested. In-text-citation has also been added on page 6.

**Reviewer’s comments**

**Reviewer 1
\# Is it necessary to add a term “prospective” in front of a “randomized double-blind trial?”**

We agree with the remark of reviewer 1 and deleted the term “prospective” in the title of the manuscript**.**

**# Some necessary components (ex: the source and number of participants, study place and period…etc.) are lacking in the abstract section**

We added all the requested information in the abstract section (see page 2).

**I suggest that the authors make comment regarding the association between “duration of dialysis” and “dysfunction of catheter” in Discussion section.**

Reviewer 1 is right to say there was a significantly longer hemodialysis vintage in the control group as compared with the TaurolockU group. This point has also been raised by reviewer three. We refer to the answers and additional analysis discussed in response to comments 1 and 2 below.

At the suggestion of reviewer 1 the potential role of dialysis vintage as a confounder of the protective effect of TaurolockU on catheter dysfunction has now been added to the discussion. (Page 26)

**Reviewer 2**

We thank reviewer 2 for the positive assessment of our study.

**Reviewer 3**

**I have three concerns:
1. The statistical analysis performed do not take account the misbalance between groups in regard of duration of dialysis. To this is required a generalized linear model.**

Reviewer 3 is right that the two groups differ in terms of duration of dialysis in spite of randomization. This is not unusual when multiple baseline characteristics are tested as on average 1 out of 20 is likely to differ “significantly” with a type I error fixed at 5%.

At the suggestion of the reviewer, we have tested whether dialysis vintage might have acted as a potential confounder of the effect of urokinase locks on the occurrence of thrombotic dysfunction. In our paper the effect of urokinase locks was tested by two types of analysis: 1) the incidence rate of thrombotic dysfunction, considering multiple episodes of thrombotic dysfunction per catheter under study and 2) the time to the first occurrence of thrombotic dysfunction in Kaplan-Meier estimates of survival free of thrombotic dysfunction. In the survival analysis catheters were censored after the first occurrence of thrombotic dysfunction.

Multivariate analysis of the incidence rate ratio after adjustment for dialysis vintage was done by Poisson regression modelling and the hazards ratio in time to event regression was realized by Cox proportional hazards regression (this part is described under question 2 of the reviewer).

The univariate IRR calculated with the STATA “poisson” command was 0.39 and thus identical the 0.39 IRR when calculating point estimates and confidence intervals for the incidence-rate ratio with the STATA “ir” command.

When dialysis vintage was introduced as a continuous variable into the model it became clear that longer dialysis vintage was associated with significantly lower incidence rates of thrombotic dysfunction. The longer dialysis vintage in the control arm therefore tended to act as a negative confounder of the association between urokinase locks and thrombotic catheter dysfunction.

After adjustment for this effect the IRR was reduced to 0.34 and remained statistically highly significant.

We have conducted the same analysis by introducing 4 quartiles of dialysis vintage as dummy variables into the regression model.

The adjusted estimate of the IRR of urokinase versus placebo was 0.32.

There was no difference in the fit of the two models based on the likelihood ratio test but slightly better fit of the model with hemodialysis vintage as a continuous data was suggested by lower values of the Akaike's and Bayesian information criterions.

We have therefore added the following phrases to the results section: *“As the control arm had a significantly longer dialysis vintage as compared to the TaurolockU arm, the incidence rate ratio was adjusted for this parameter by Poisson regression modelling. The univariate IR ratio for TaurolockU as only variable in the model was 0.39 (95%CI 0.24 to 0.63; P<0.0001). Longer dialysis vintage was associated with a significantly lower incidence of thrombotic dysfunction (IR ratio 0.93 per year (95%CI 0.87 to 0.99; P=0.04). After adjustment for dialysis vintage the adjusted IR ratio for TaurolockU use was 0.34 (95%CI 0.21 to 0.56; P<0.0001).”* (Page 16)

**2. In a cohort in which outcome may occur at many points of time, there is a need for Cox analysis regression that compares the hazard risk of outcome (catheter thrombosis), take account the time interval between the time zero to the event, and make survival curves adjusted to the misbalances between groups. In this case, bivariate analysis will be sufficient because groups were heterogeneous only in regard of the duration of dialysis.**

The use of urokinase was associated with a univariate Cox hazard ratio for thrombotic catheter dysfunction of 0.43 (95%CI 0.22 to 0.84).

Adding dialysis vintage as a continuous variable reduced the hazards ratio to 0.37.

Adding hemodialysis vintage as dummy variables of quartiles reduced the hazards ratio to 0.31.

The proportional hazards assumption was respected for the two models. The likelihood of the two models did not differ significantly when tested by the likelihood ratio test. The model with hemodialysis vintage as a continuous data was chosen because it had slightly lower values of the Akaike's and Bayesian information criterions.

The following phrase was therefore added to the paper: *“The univariate Cox proportional hazards ratio for the use of urokinase locks was 0.43 (95%CI 0.22 to 0.84; P=0.013). When dialysis vintage was added to the Cox model the hazards ratio was 0.37 (95%CI 0.18 to 0.74; P=0.005).”* (Page 16)

We are grateful to reviewer 3 to have raised the issue of a possible confounding role of dialysis vintage on the reduction of thrombotic dysfunction by TaurolockU locks. Following the suggestion of reviewer 1 this has now been added in the discussion to the limitations of the paper.

*"Fourth, in spite of randomization the control group had a longer time on dialysis therapy at the moment of inclusion into the study. However, longer dialysis vintage was associated with a lower risk of thrombotic dysfunction. This variable therefore acted as a negative confounder of the protective effect and adjustment for dialysis time further lowered the incidence rate ratio of thrombotic catheter dysfunction with the use of TaurolockU."* (Page 26)

**2. Author should take into account the cost of waste of time to make thrombolysis in a catheter in the cost analysis.**

This is a correct criticism. We did not record the time nurses spent on treating dysfunctional catheters which includes application of therapeutic locks but also flushing of catheters and aspiration of thrombi. This shortcoming has been added to the discussion.

*"Fifth, the cost analysis of the intervention was limited to the cost of the two prophylactic catheter locks and of therapeutic urokinase locks. This unfortunately does not take into account additional cost such as the time nurses spent to treat occluded or dysfunctional catheters."*

We consider that the revisions in response to the comments of the reviewers have improved the quality of our paper and hope that it can be considered at present suitable for publication in PlosOne.

Sincerely yours,

Prof Karl Martin Wissing
